# Mycobacterial respiratory chain enzymes and growth are inhibited by decylubiquinone

**DOI:** 10.1038/s42003-025-09309-9

**Published:** 2025-12-10

**Authors:** Sylwia Król, Terezia Kovalova, Mateusz Janczak, Sadaf Kalsum, Mira Akber, Martin Högbom, Susanna Brighenti, Pia Ädelroth, Peter Brzezinski

**Affiliations:** 1 Department of Biochemistry and Biophysics, The Arrhenius Laboratories for Natural Sciences, Stockholm University, Stockholm, Sweden; 2 Division of Medical Microbiology and Molecular Medicine, Department of Clinical and Experimental Medicine, Linköping University, Linköping, Sweden; 3 Center for Infectious Medicine (CIM), Department of Medicine Huddinge, Karolinska Institutet, ANA Futura, Huddinge, Sweden

**Keywords:** Cryoelectron microscopy, Bioenergetics, Oxidoreductases

## Abstract

Aerobic organisms obtain energy by linking electron transfer from NADH to O_2_, through the respiratory chain, to transmembrane proton translocation. In mycobacteria the respiratory chain is branched; the membrane-bound electron carrier menaquinol (MQH_2_) donates electrons either to the O_2_-reducing cytochrome *bd* or a supercomplex that is composed of a complex (C) III_2_ dimer flanked by two CIVs. Here, we measured the dimethyl-naphthoquinone (DMNQH_2,_ a menaquinol analogue) oxidation:O_2_ reduction activities of the CIII_2_CIV_2_ supercomplex and cytochrome *bd* in the presence of an analogue (decylubiquinone, DCQ) of the mammalian electron carrier, ubiquinol. The data show that DCQH_2_ inhibits both the CIII_2_CIV_2_ and cytochrome *bd* activities, suggesting that DCQ/DCQH_2_ interferes with both branches of the respiratory chain. Cryo-EM data of the *M. smegmatis* supercomplex shows that oxidized DCQ binds in the electron donor site (Q_o_) of CIII_2_. Accordingly, growth of *M. smegmatis* cells was impaired in the presence of DCQ. Remarkably, DCQ also impairs intracellular growth of virulent *M. tuberculosis* cells in human primary macrophages suggesting that the compound could potentially be used as an adjuvant during tuberculosis disease treatment.

## Abstract (web summary)

Using a combination of cryo-electron microscopy, activity measurements and in vivo experiments, the authors show that decylubiquinone, an analogue of the mammalian electron carrier, ubiquinol, binds to and inhibits the CIII_2_CIV_2_ supercomplex from *M. smegmatis*, and inhibits growth of *M. tuberculosis*. This compound may thus represent a new therapeutic strategy to treat tuberculosis.

## Introduction

In the last steps of aerobic respiration, exergonic electron transfer drives proton translocation or pumping across the mitochondrial inner membrane or bacterial cytoplasmic membrane to maintain a transmembrane proton electrochemical gradient. Free energy stored in this gradient is used to drive transmembrane transport processes and the synthesis of ATP. In mammalian mitochondria, electron-transport chain complexes (C) I and II reduce the membrane-bound two-electron carrier ubiquinone (UQ), also known as coenzyme Q, to ubiquinol (UQH_2_), which donates electrons to dimeric CIII_2_, also known as cytochrome *bc*_1_. Complex III is an electron donor to cytochrome *c*, which reduces CIV (cytochrome *c* oxidase) that transfers electrons to molecular oxygen, which is reduced to water at a heme-copper catalytic site (for recent reviews, see e.g., refs. ^1–3^).

**Fig. 1:**
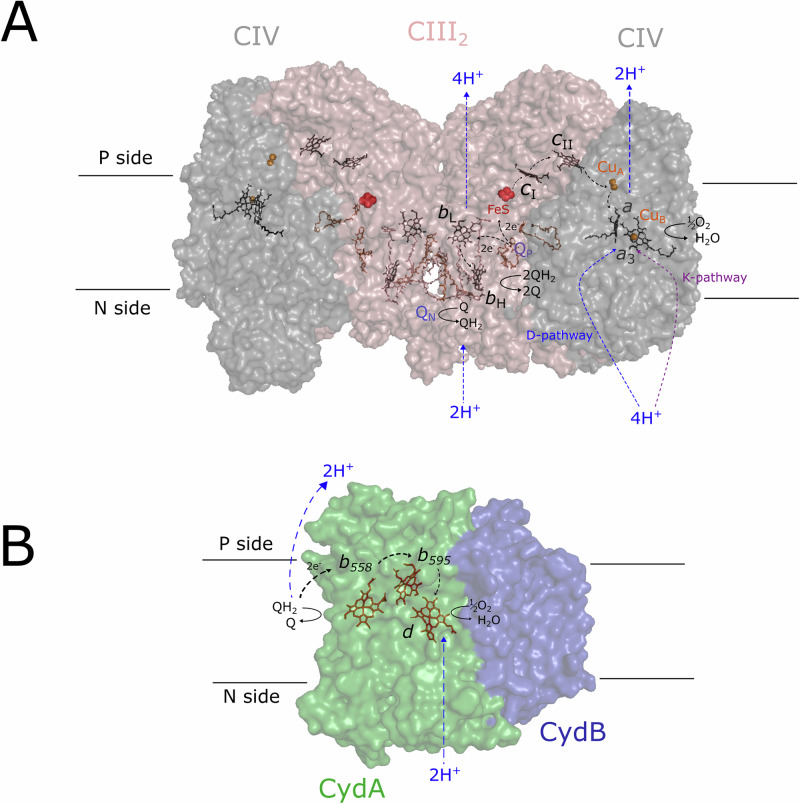
Overview of the studied complexes. Overall structures of the CIII_2_CIV_2_ supercomplex (**A**) and cytochrome *bd*-I (**B**). Electron and proton-transfer pathways are indicated with black and colored dashed arrows, respectively. The negatively and positively charged sides of the membrane are indicated by N and P, respectively. The *M. smegmatis* CIII_2_CIV_2_ supercomplex and cytochrome *bd*-I structural models are PDB ID 6ADQ^11^ and 7D5I^77^, respectively.

In CIII_2_, UQH_2_ binds in a quinone-binding site called Q_o_ (also called Q_P,_ Fig. 1A). Crystal structures of canonical CIII_2_s have the Q_o_ site typically empty and its position was originally suggested based on observation of binding of Q_o_-site inhibitors such as myxothiazol or stigmatellin^4,5^. More recent cryo-EM data revealed endogenous UQ in the Q_o_ sites in CIII_2_, e.g., from the pathogenic yeast *C. albicans*^6^ and in one monomer of CIII_2_ in a mammalian CI-CIII_2_ supercomplex^7^. In canonical CIII_2_ the UQ benzoquinone head group is located deeply inside the Q_o_ site cavity to allow oxidation of UQH_2_ by the Rieske head domain. Density for an endogenous menaquinone (MQ) was found at the same position in the Q_o_ site as in canonical CIII_2_ (Q_o1a_) in the actinobacterial *C. glutamicum* CIII_2_^8,9^. However, MQ in the *M. smegmatis* CIII_2_ occupies a more distal position in the Q_o_ site, referred to as Q_o1b_, at the entrance to the Q_o_ site cavity, with the naphthoquinone head group found ~15 Å from the FeS cluster electron acceptor^10,11^.

The quinol is oxidized in a branched electron-transfer reaction referred to as the Q-cycle (for review, see refs. ^5,12^). One electron is transferred from QH_2_ to a Rieske iron-sulfur site (FeS), which transfers electrons consecutively to cytochrome *c*_1_ and a water-soluble cytochrome *c* that docks near cytochrome *c*_1_. The second electron is transferred sequentially to hemes *b*_L_, *b*_H_ and then to a Q bound in the Q_i_ (also called Q_N_) site, which forms a semiquinone. Protons are released from QH_2_ in the Q_o_ site to the membrane positive (*p*) side. Upon binding of a second UQH_2_ in the Q_o_ site, the same sequence of reactions is repeated, leading to reduction of the semiquinone in the Q_i_ site, uptake of two protons from the membrane negative (*n*) side and formation of QH_2_, which equilibrates with the Q/QH_2_ pool in the membrane. The overall Q-cycle process results in a transmembrane charge separation that is equivalent to the translocation of one proton from the *n* to the *p* side of the membrane for each electron that is transferred from QH_2_ to cytochrome *c*. In CIV, electrons are initially transferred from cytochrome *c* to a copper center, Cu_A_, and then consecutively to heme *a* and the heme *a*_3_-Cu_B_ catalytic site, which, in its reduced state, binds O_2_. Reduction of O_2_ to H_2_O is linked to proton uptake from the *n* side of the membrane, which also in CIV results in a charge separation across the membrane that is equivalent to the transfer of one positive charge across the membrane for each electron transferred from cytochrome *c* to O_2_. In addition, each electron transfer to the catalytic site is linked to proton pumping across the membrane, which doubles the charge-separation stoichiometry (for review, see refs. ^13–16^). In the CIII_2_CIV_2_-mediated menaquinol (MQH_2_) oxidation pathway in *M. smegmatis*, MQH_2_ binds near the FeS domain in each monomer of CIII_2_ (Fig. 1A), and electrons are presumably transferred utilizing a Q-cycle mechanism, similar to that in the mitochondrial CIII_2_^17^. Mycobacteria do not harbor a water-soluble cytochrome *c* and electrons from the FeS site are instead transferred to Cu_A_ via a cytochrome *cc* domain of each monomer of CIII_2_^18^.

Mycobacteria, including the human pathogen *Mycobacterium tuberculosis*, belong to the phylum Actinobacteria. These Gram-positive bacteria utilize aerobic respiration to extract energy from various carbon sources during growth^19,20^. In contrast to eukaryotes and many other bacteria, the electron-transfer chain in mycobacteria is exceedingly branched^20^. In the last steps of the mycobacterial respiratory chain, electrons from MQH_2_ are transferred to O_2_ through two pathways that involve different types of oxidoreductases: cytochrome *bd*-type quinol oxidases and a heme-copper *aa*_3_-type CIV, which is part of an obligate CIII_2_CIV_2_ supercomplex (Fig. 1A). Cytochrome *bd* oxidases are found only in prokaryotes where they catalyze oxidation of QH_2_ and reduction of O_2_ to H_2_O, but they are evolutionarily unrelated to heme-copper oxidases (for a recent review, see ref. ^21^). The *bd* oxidases do not pump protons and energy conservation arises only from a transmembrane charge separation upon proton release from QH_2_ to the *p* side and proton uptake upon O_2_ reduction from the *n* side, linked to intramolecular electron transfer. The enzymes harbor three heme groups, two heme *b*s (termed *b*^558^ and *b*^595^) and a heme *d*, which is the site of reduction of O_2_ to H_2_O. The relative position of heme *b*^595^ and heme *d* varies between organisms and their involvement in electron transfer and catalysis is not fully understood^1,21^. Structures of the *E. coli* cytochrome *bd*s^22–24^ and actinobacterial cytochrome *bd*s (Fig. 1B)^25–27^ are overall similar and suggest that electrons from QH_2_ are initially transferred consecutively to heme *b*^558^, heme *b*^595^ and heme *d*. *M. smegmatis*, like *E. coli*, harbors two cytochrome *bd* gene clusters, the *cydAB* (*bd*-I) and *appBC* (*bd*-II)^28,29^, but evidence for a biochemical role exists only for *bd*-I. Analysis of the cytochrome *bd* structures suggests variability among the QH_2_ binding sites in *E. coli* and also among different actinobacteria^21,22,24–27,30^.

MQ has a lower midpoint potential (*E*_m7_ ≅ −80 mV^31,32^) than the mammalian electron carrier, UQ (*E*_m7_ ≅ +100 mV^31,33^). Some Gram-negative bacteria are known to exchange between UQ and MQ depending on oxygen concentration. This switch between different isoprenoid quinones does not take place in Gram-positive bacteria^31,34^. On the other hand, it has been shown that mycobacteria maintain cellular bioenergetics in oxygen-deficient habitats such as biofilms by switching to the synthesis of polyketide quinones^35^. Because under low-oxygen concentrations *M. smegmatis* switches from using the *aa*_3_-type heme-copper CIV to cytochrome *bd*^36^, the polyketide quinone is presumably used as an electron donor to the latter^35^.

A number of drug candidates that target oxidative phosphorylation in *M. tuberculosis* have been identified. These drugs inhibit selectively either electron transfer to O_2_ or the ATP synthase^37^, thereby impairing ATP synthesis. Bedaquiline inhibits mycobacterial ATP synthase^38^ by inducing structural changes that result in tight binding at the interface of two of the enzyme’s subunits^39^. The electron-transport chain branch through CIII_2_CIV_2_ is inhibited by telacebec (Q203)^40^ or Lansoprazole (LPZ) metabolized to its sulfide form (LPZS)^41^ by competing with MQ/MQH_2_ binding to the Q_o_ site in CIII_2_^10,42^. However, killing of *M. tuberculosis* may require blocking both the CIII_2_CIV_2_ and cytochrome *bd* electron transfer branches to O_2_^43,44^. In the current study, we investigated oxidation of a menaquinone analog, dimethyl-naphthoquinone (DMNQH_2_), by O_2_, catalyzed by either the isolated *M. smegmatis* cytochrome *bd*-I or CIII_2_CIV_2_ supercomplex in the presence of the UQH_2_ analog decylubiquinone (DCQ). Kinetic data show that both CIII_2_CIV_2_ and cytochrome *bd*-I are inhibited by DCQ, and cryo-EM data reveal that DCQ binds to the Q_o_ site in CIII_2_. ^44^Furthermore, we show that DCQ inhibits the growth of *M. smegmatis* cells and impairs the intracellular growth of virulent *M. tuberculosis* in human primary macrophages, suggesting that DCQ could be explored as a potential drug targeting pathogenic mycobacteria.

## Results and discussion

### Naphthoquinol oxidation activity of the isolated CIII_2_CIV_2_ supercomplex

We measured the quinol oxidation: O_2_ reduction activity of the *M. smegmatis* CIII_2_CIV_2_ supercomplex by monitoring the oxygen-reduction rate upon addition of CIII_2_CIV_2_ to a solution containing reduced 2,3-dimethyl-[1,4] naphthoquinone (DMNQH_2_), which is used as an analog of MQ. The maximum supercomplex activity was measured at 100 μM DMNQH_2_ and was found to be 80 ± 10 s^−1^ (one measurement on each of three different supercomplex preparations).

### DMNQH_2_ oxidation activity of the isolated cytochrome *bd*-I

The oxidoreductase activity of the *M. smegmatis* cytochrome *bd*-I was observed by measuring the oxygen-reduction rate upon addition of cytochrome *bd*-I to a solution containing DMNQH_2_. As described above, we monitored the O_2_-reduction rate before the addition of cytochrome *bd*-I, and this slope was subtracted from that obtained after the addition of cytochrome *bd*-I. The activity observed at 300 μM DMNQH_2_ was 18 ± 1 s^−1^ (*n* = 3).

### Activities of isolated CIII_2_CIV_2_ and cytochrome *bd*-I in the presence of decylubiquinone

**Fig. 2:**
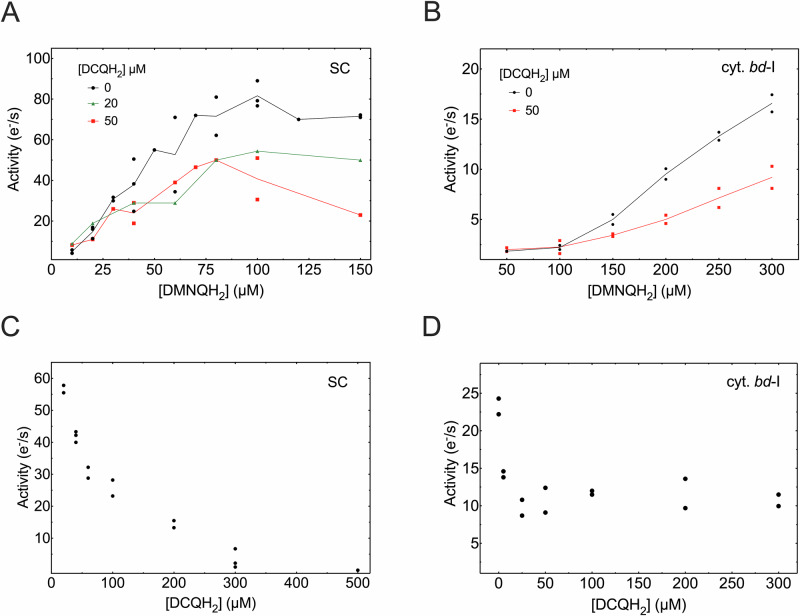
Supercomplex and cytochrome *bd*-I activities. CIII_2_CIV_2_ supercomplex (**A**) and cytochrome *bd*-I (**B**) activity as a function of the DMNQH_2_ concentration, in the absence and presence of DCQH_2_. Supercomplex (**C**) and cytochrome *bd*-I (**D**) activity as a function of the DCQH_2_ concentration, with 100 μM MQH_2_. All measured values are shown in the figure. Where applicable, the solid line is drawn through the average of the measured values.

We used decylubiquinone (DCQ), which is an ethanol-soluble, commonly used (e.g., ref. ^45^) analog of UQ^46^. The CIII_2_CIV_2_ supercomplex activity with reduced DCQ was ≤2 s^−1^, i.e., ≤3% of the maximum activity measured with DMNQH_2_, both at a concentration of 100 μM. The activity of cytochrome *bd*-I with DCQ was too low to be measured. Figure 2A shows the DMNQH_2_ oxidation: O_2_ reduction activity of the supercomplex as a function of the DMNQH_2_ concentration. As seen in the figure, the activity increased with increasing DMNQH_2_ concentration up to ~100 μM DMNQH_2_ and then decreased slightly above 100 μM DMNQH_2_. In the presence of DCQH_2_ at 20 μM or 50 μM, in general, a lower turnover rate was observed. In addition, with 50 μM DCQH_2_, a decrease in activity at DMNQH_2_ concentrations >100 μM was observed. The reason for this decrease is unknown, but we note that under native conditions the Q-cycle of CIII_2_ involves oxidation of MQH_2_ at the Q_o_ site as well as reduction of MQ the Q_i_ site. In other words, during turnover, two different sites compete for Q/QH_2_ and most likely display different binding constants for the different redox states and the two different quinones, giving potentially rise to complicated kinetics.

Figure 2B shows the DMNQH_2_ oxidation: O_2_ reduction activity of cytochrome *bd*-I as a function of DMNQH_2_ concentration. As seen in the figure, the activity increased with increasing DMNQH_2_ to reach ~18 s^−1^ at 300 μM DMNQH_2_. This activity would presumably increase further above 300 μM DMNQH_2_, but these measurements were precluded by the too low solubility of DMNQH_2_. In the presence of 50 μM DCQH_2_, the DMNQH_2_-oxidation activity was slower and at 300 μM it was ~45% of that in the absence of DCQH_2_ (Fig. 2B).

Figure 2C shows the supercomplex activity measured with 50 μM DMNQH_2_ as a function of added DCQH_2_. As seen in the figure, the activity dropped, with an IC_50_ of ~70 μM, to reach ~3% of the maximum activity at 500 μM DCQH_2_. Also, with cytochrome *bd*-I, the activity dropped upon addition of an increasing amount of DCQH_2_ (Fig. 2D), but at a saturating concentration of ~20 μM DCQH_2_, it remained at ~10 s^−1^, i.e., ~42% of the maximum activity.

In the measurements described above, the inhibitory DCQ was added in the reduced form, i.e., DCQH_2_. Because the midpoint potential of DCQ/DCQH_2_ is higher than that of DMNQ/DMNQH_2_, in the presence of DCQ, any added DMNQH_2_ would otherwise be oxidized directly by DCQ.

### Modulation of DCQH_2_ inhibition by engineering the Q_o_ site

**Fig. 3:**
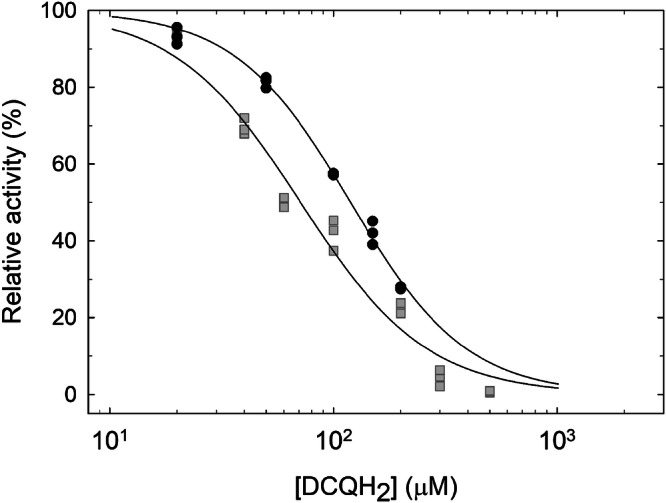
Activity of the wild-type (WT, grey squares) and M337A, M305A, F153A triple (referred to as AAA, black circles) mutant supercomplex as a function of the concentration of added DCQH_2_. The DMNQH_2_ concentration was 100 μM. The solid line is a fit of the data with the equation: $\theta =\left({1+\left(\displaystyle \frac{{{{\rm{K}}}}_{{{\rm{A}}}}}{\left[{{\mathrm{DCQH}}}_{2}\right]}\right)}\right)^{-1}$ where *θ* is the percent fraction maximum activity, *K*_A_ is the DCQH_2_ concentration at which half the maximum rate is obtained, [DCQH_2_] is the concentration added DCQH_2_. The maximum activity in the absence of added DCQH_2_ is 80 s^−1^ and 50 s^−1^ for the WT and AAA variant, respectively. The supercomplex activity with 100 μM DCQH_2_ was ≤3% of the maximum activity with 100 μM DMNQH_2_, therefore, the two solid lines are assumed to approach zero at the highest DCQH_2_ concentrations.

To further analyze the inhibitory effect of DCQH_2_, we used a structural variant of CIII_2_ in which residues near the Q_o_ site were modified^42^: the M337A, M305A, F153A triple mutation of the QcrB subunit^42^. The structural variant (termed AAA) was expressed and purified using the same approach as that used with the wild-type supercomplex. The DMNQH_2_:O_2_ oxidoreduction activity of the structural variant was ~50 s^−1^ (at 100 μM DMNQH_2_), i.e., approximately 60% of the wild-type activity. Titration of the CIII_2_CIV_2_ supercomplex activity as a function of added DCQH_2_ showed that for the triple mutant, the IC_50_ increased from ~70 μM (wild-type) to ~130 μM (Fig. 3), suggesting that DCQH_2_ binds at the same site as DMNQH_2_.

### Reduced growth of *M. smegmatis* in the presence of DCQ

**Fig. 4:**
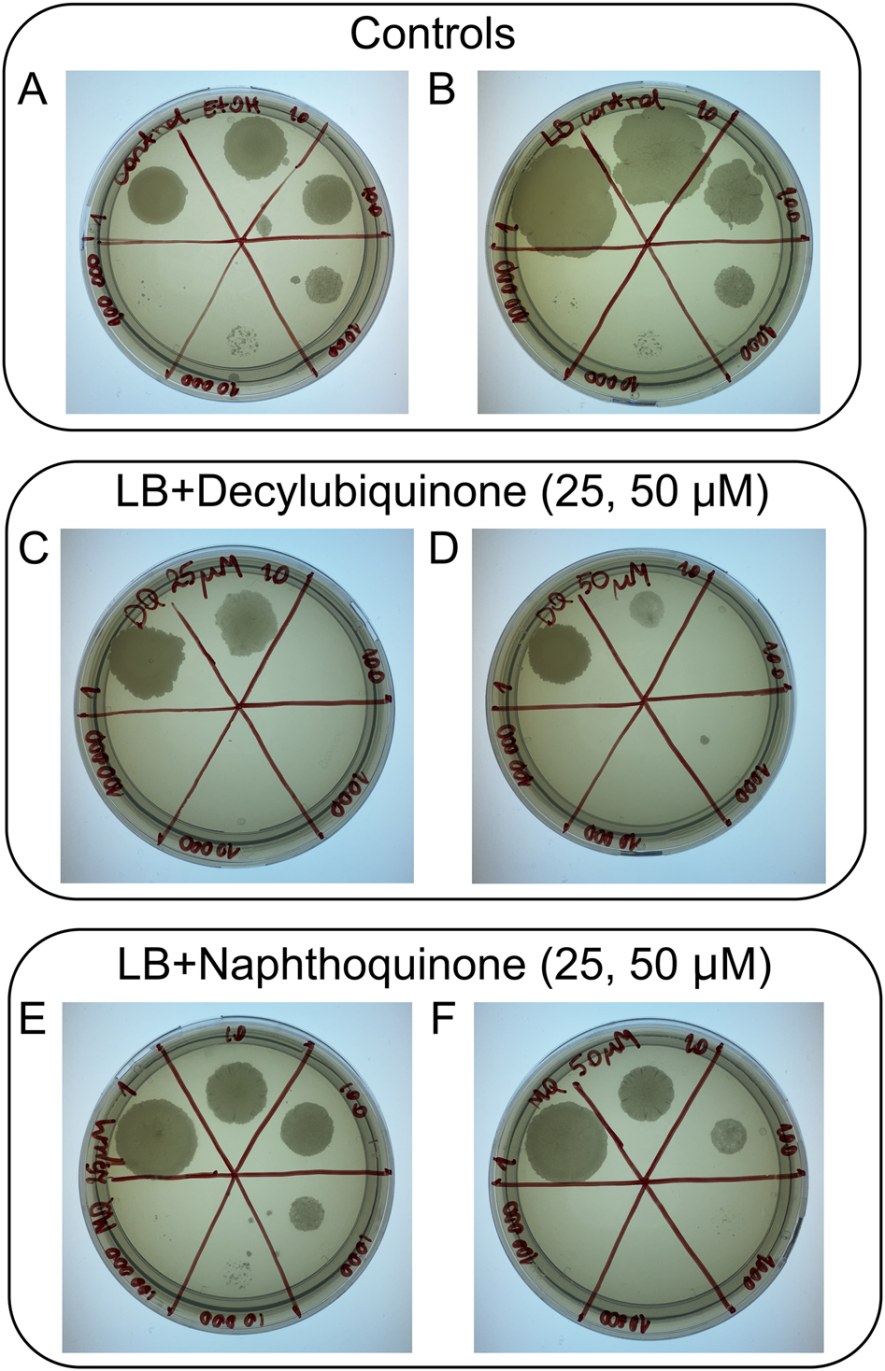
Growth of *M. smegmatis* cells in the presence of DCQ or DMNQ. **A** Control plate with 75 μl 98% ethanol, added to the liquid agar before it set. **B** Control plate without any additions. **C** 25 μM or **D** 50 μM decylubiquinone (DCQ) added. **E** 25 μM or **F** 50 μM naphthoquinone (DMNQ) added. Each plate was divided into six parts where each part represents a different cell dilution with LB by factors, from “1” (undiluted) to “100,000”.

To test the effect of DCQ or DMNQ on the growth of *M. smegmatis*, cells were plated on LB medium + kanamycin, supplemented with different concentrations of DCQ or DMNQ. In Fig. 4, each section of each plate represents different bacterial culture dilutions, marked with numbers ranging from 1 (undiluted) to 100,000. As observed in panels Fig. 4C and D, addition of DCQ resulted in impaired bacterial growth. Addition of 25 µM DMNQ did not exhibit any effect, while at 50 µM DMNQ, growth was somewhat impaired at bacterial culture dilutions by a factor of >1000 (Fig. 4E, F).

### Reduced growth of virulent *M. tuberculosis* cells in human macrophages upon treatment with DCQ

**Fig. 5:**
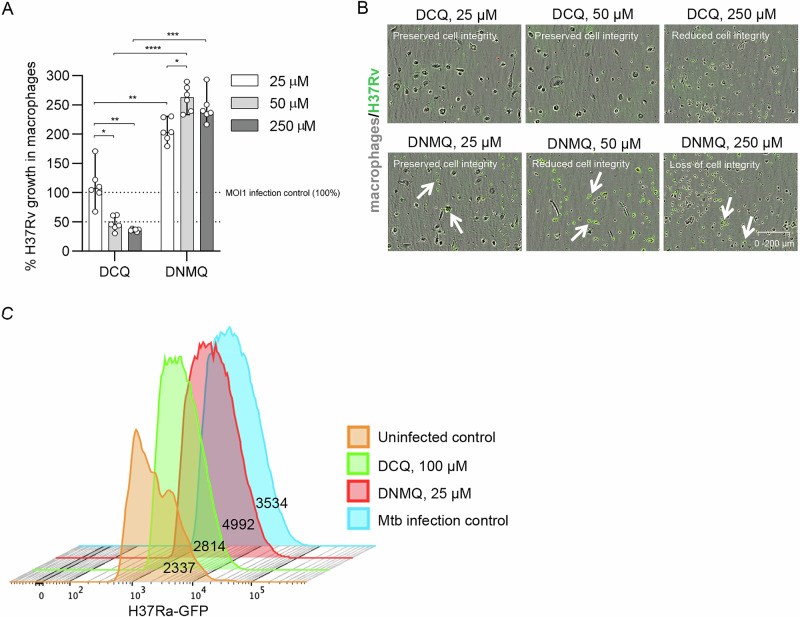
Efficacy of DCQ and DMNQ to reduce growth of *M. tuberculosis* in primary human macrophages. **A** High-content analysis with IncuCyte at day 4 was used to quantify intracellular H37Rv growth in infected macrophages treated with 25, 50, or 250 µM doses of DCQ or DMNQ compared to the MOI1 infection control. The dotted lines indicate the MOI1 infection control set to 100% growth, as well as 50% Mtb growth in macrophages. Statistical differences comparing different doses of compounds (median ± range) were determined using a 2-way ANOVA and Tukey’s multiple comparisons test. ****p < 0.0001, ***p < 0.001, **p < 0.01, *p < 0.05. One representative experiment out of three similar, including *n* = 6 replicates from two donors, is shown. Data are displayed in a dot-and-bar plot showing median and range. **B** Microscopy images showing macrophages (light gray color) infected with H37Rv-GFP (green color) and treated with different concentrations of DCQ (upper panel) or DMNQ (lower panel). Samples from whole well images, magnification ×20. Observe the changes in morphological features as the concentrations of UDCQ and DMNQ increase, ranging from preserved cell integrity (characterized by relatively large, irregular cells with ovoid nuclei) to reduced or lost cell integrity (evidenced by small, rounded, shrunken, and condensed cells). Arrows indicate H37Rv-GFP infected cells that were numerous upon DMNQ treatment. **C** Flow cytometry was used to assess intracellular H37Ra bacteria in primary human macrophages after 48 h treatment with DCQ and DMNQ using concentrations that maintain cellular integrity. Results were compared to MOI5 infection control as well as the uninfected control. The geometric mean of the GFP-positive (+) subset gated on live cells is shown in the histogram. Mtb *M. tuberculosis*.

Next, the potency of DCQ to reduce growth of virulent *M. tuberculosis* expressing green fluorescent protein (GFP) was explored using a well-established macrophage infection model and high-content imaging^47^. As mycobacteria are facultative intracellular pathogens, this represents a physiological model to study inhibition of intracellular growth. Different concentrations of DCQ and DMNQ were tested for the ability to inhibit intracellular growth of H37Rv in human monocyte-derived macrophages after 4 days culture (Fig. 5A). Accordingly, we observed a dose-dependent reduction of H37Rv growth in the presence of DCQ, while there was a corresponding increase of mycobacterial growth in the presence of DMNQ (Fig. 5A). The antimycobacterial effect of DCQ at 50 µM and 250 µM was significantly higher compared to 25 µM (*p* < 0.016 and *p* < 0.0054, respectively). Instead, there was a significantly increased growth of H37Rv upon treatment with 25 µM, 50 µM and 250 µM DMNQ (*p* < 0.0094–0.0001) as compared to the corresponding concentrations of DCQ (Fig. 5A). Lower concentrations of DCQ (1 and 10 μM) did not significantly affect intracellular *M. tuberculosis* growth compared to the MOI infection control. Overall, DCQ-mediated growth inhibition of H37Rv at doses of 50 µM and 250 µM (53–64%) was also significant compared to the MOI1 infection control (*p* < 0.003–0.0001), while there was no significant difference between the 25 µM DCQ treatment and the MOI1 infection control (Fig. 5A). In contrast, H37Rv growth was significantly enhanced by different concentrations of DMNQ (*p* < 0.041–0.0001).

Microscopic examination of *M. tuberculosis*-infected macrophages revealed altered cellular morphology at higher concentrations of DCQ and especially DMNQ, including rounded and shrunken cells (Fig. 5B). These changes could indicate incipient cytotoxicity induced by higher doses of the compounds. Notably, macrophage morphology was preserved at DCQ doses of 25 and 50 µM (Fig. 5B, upper panel), while DMNQ treatment reduced cellular integrity even at the lower dose of 50 µM (Fig. 5B, lower panel). Despite the compromised morphology of *M. tuberculosis*-infected cells at the higher 250 µM dose (Fig. 5B, upper panel), DCQ clearly maintained its antimycobacterial activity (Fig. 5A), indicating direct effects on the bacteria and the ability to inhibit both intra- and extracellular *M. tuberculosis* growth.

Consistent with the high-content imaging results (Fig. 5A, B), flow cytometry analysis quantifying avirulent H37Ra-GFP inside human macrophages confirmed that DCQ could inhibit mycobacterial growth at 48 h (Fig. 5C). This effect was observed comparing DCQ (geometric mean (GM): 2814) to both DMNQ (GM: 4992) and the untreated infection control (GM: 3534), using compound doses that maintained cell integrity (Fig. 5C). Altogether, these data suggest that DCQ possesses potent antimycobacterial activity, whereas mycobacteria may exploit DMNQ to enhance their survival in host cells.

### Cryo-EM structure of the CIII_2_CIV_2_ supercomplex with bound DCQ

**Fig. 6:**
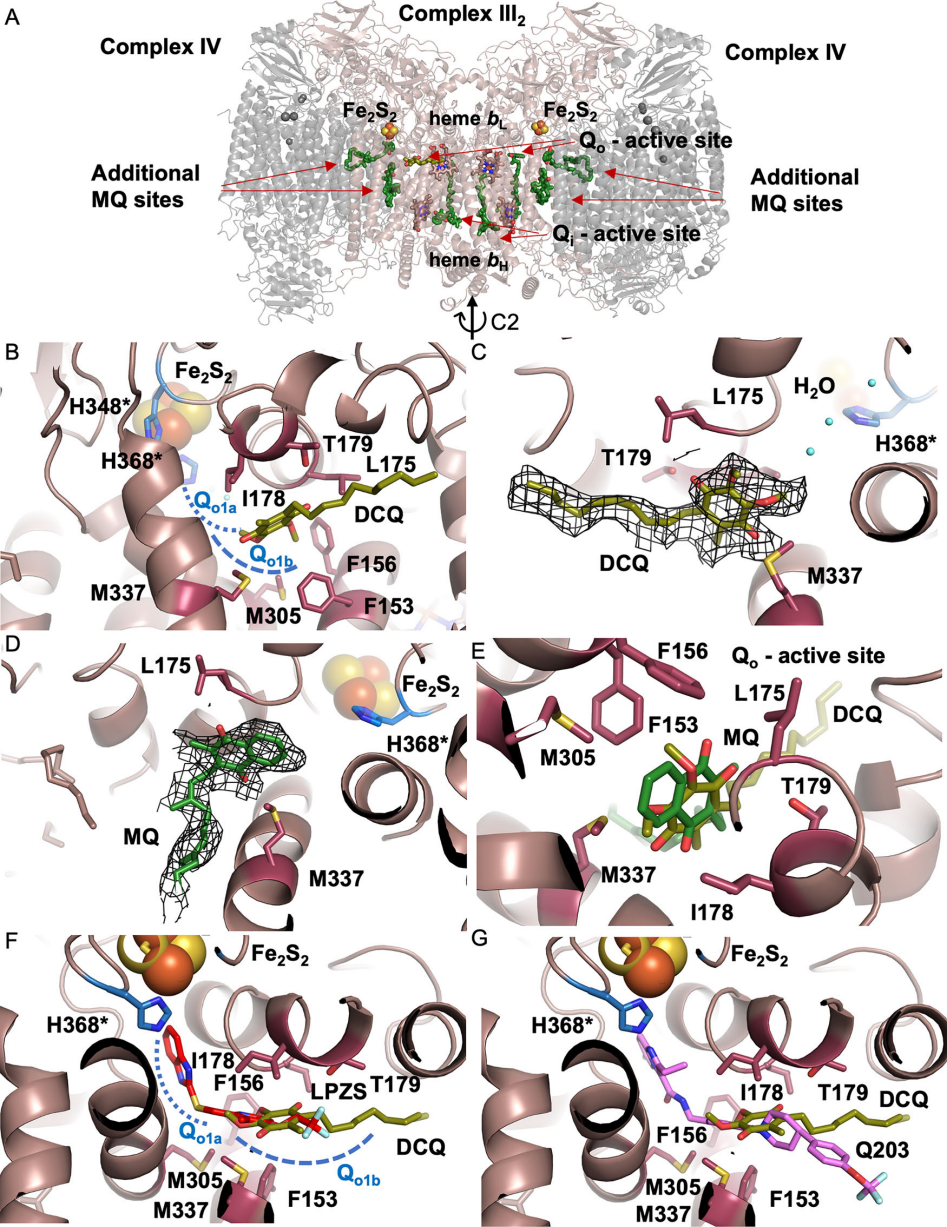
Structure of the supercomplex with bound DCQ. **A** MQ-binding active sites Q_o_ and Q_i_, respectively, and additional binding sites in CIII_2_ (pink) of the supercomplex. The two subsites, Q_o1a_ and Q_o1b_, of the of Q_o_ site are marked with a blue dashed line. MQ and DCQ in its binding site (Q_o1b_ position) are shown in dark and light green, respectively. **B** Q_o_ cavity with bound DCQ (light green) and interacting residues. Residues from the Rieske subunit are marked by an asterisk. **C** Comparison of the binding poses of MQ and DCQ in the Q_o1b_ binding site. **D**, **E** Comparison of the binding of DCQ and known Q_o_ site inhibitors, lansoprazole sulfide (**D**, red^42^) and Q203 (**E**, pink^10^). The panels are an overlay of the current structure and those published earlier with these two inhibitors. Cryo-EM electron density maps of the DCQ (**F**) or MQ (**G**) in the Q_o1b_ site of the supercomplex (*σ* = 2 level).

In order to verify the binding site of DCQ in CIII_2_, we determined a cryo-EM structure of the *M. smegmatis* CIII_2_CIV_2_ supercomplex solubilized in GDN, which was incubated with DCQH_2_ in the presence of O_2_ prior to cryo-EM grid preparation. The structure of the supercomplex was solved to an overall resolution of 2.5 Å without symmetry (C1) (Fig. 6A). Its architecture and subunit composition agree with already published *M. smegmatis* supercomplex structures^11,48,49^. The complex is composed of centrally positioned CIII_2_ dimer where each CIII monomer consist of three main functional subunits: QcrB, which harbors two cytochrome *b*s and the quinol/quinone binding sites, QcrA Rieske subunit, which harbors the FeS iron-sulfur cluster and the membrane-bound cytochrome *cc* subunit (QcrC) with two heme *c* containing domains. One of these domains is attached to the Rieske subunit, the other is in direct interface with CIV. The cytochrome *cc* subunit creates an electron-transfer bridge to the two copies of CIV on each side of the CIII_2_, forming a direct path for electron transfer from CIII to CIV. CIV is composed of main functional subunits CtaC, which harbors a dinuclear Cu_A_ and CtaD with heme *a* and the heme *a*_3_-Cu_B_ binuclear active site, where O_2_ is reduced to H_2_O. Additional CIV subunits are CtaE and CtaF and actinobacteria-specific CtaI and CtaJ. Accessory subunits LpqE and AscA stabilize the connection between CIII_2_ and CIV. In addition, a superoxide dismutase is part of the supercomplex.

As mentioned above, there are two substrate binding sites in each CIII monomer, an electron donor site Q_o_ where the quinone can adopt a proximal Q_o1a_ position and a more distal Q_o1b_ position towards FeS center (Fig. 6B), and an electron acceptor site Q_i_. Because CIII_2_ is a dimer, there are two copies of each quinone-binding site. The cryo-EM data identified densities corresponding to substrate quinones bound at each Q_o_ site. However, the density in one monomer of the CIII_2_ dimer could be interpreted as the externally added DCQ (Fig. 6C) while in the other site it was more similar to the native MQ (Fig. 6D), which is typically copurified with the supercomplex. Hence, the densities are most likely a combination of both molecules. For this reason, a 50% occupancy was assigned to each of MQ and DCQ. The DCQ density is seen in the more distal Q_o1b_ position, i.e., the same as that of the native MQ (see superposition of the two quinones, DCQ and MQ, in Fig. 6E), in accord with the earlier observations^10,11^. MQ and DCQ display the same orientation in the binding site, with the aromatic head pointing towards the Q_o1a_ site and the aliphatic tail placed outside the cavity in the membrane space. After binding at the Q_o1b_ position, the native MQH_2_ presumably moves to the more proximal Q_o1a_ position (c.f. Fig. 6B) to transfer an electron and proton to the FeS Rieske domain. The inhibitory action of DCQ is explained by competition of DCQ and MQ for the same site, where DCQ would be unable to move to Q_O1a_ or adopt a correct orientation to transfer an electron to FeS. In Fig. 6B–D, some residues that define the MQ_o1b_ site are shown; Phe153, Phe156, Met305, Met337 and Leu 175, form hydrophobic interactions with the substrate quinones, and a possible hydrogen bond is formed with Thr179. Because the sample was prepared under aerobic conditions, the oxidized state is more likely and, therefore, both MQ and DCQ are modeled in their oxidized states.

Structures of CIII_2_CIV_2_ with bound lansoprazole sulfide (LPZS)^42^ or telacebec (Q203)^10^ indicate that these inhibitors block MQH_2_ binding to both Q_o1a_ and Q_o1b_ sites^37^. Figure 6FG shows an overlay of DCQ (shown in yellow) along with LPZS (panel F, red) and telacebec (panel G, pink).

In earlier studies, the electron density at the Q_i_ site was found to be too disordered for distinction of the orientation of the MQ headgroup, however, the tail part of the molecule was clearly resolved. In the current structure, the situation is very similar, while it is difficult to resolve the headgroup, there is a density for the aliphatic tail, which is consistent with the native MQ (menaquinone-9, with nine isoprene units^50^). In other words, it is unlikely that DCQ is bound in the Q_i_ site.

As observed earlier, a number of additional ligands were resolved in the supercomplex structure, including extra MQ-binding sites, with unknown function (see Fig. 6A)^10,11^, together with multiple cardiolipin molecules located in cavities between CIII_2_ and CIV.

The combined cryo-EM and kinetic data indicate competitive binding of DCQH_2_ and DMNQH_2_ to the same Q-binding site. The dual action of DCQ, impairing both CIII_2_CIV_2_ and the cytochrome *bd* branches of the electron-transport chain, makes this compound a promising inhibitor of mycobacterial respiration, which, in combination with e.g., Bedaquiline, could potentially synergize to abolish oxidative phosphorylation^44^.

### Summary and conclusions

Oxidation of both UQH_2_ (*E*_m7_ ≅ +100 mV^31,33^) and MQH_2_ (*E*_m7_ ≅ −80 mV^31,32^) catalyzed by the *M. smegmatis* CIII_2_CIV_2_ supercomplex or cytochrome *bd*-I is a highly exergonic reaction because the reduction of the final electron acceptor, O_2_, to H_2_O has an *E*_m7_ = + 820 mV^51^. However, for the supercomplex, the kinetics of the QH_2_ oxidation: O_2_ reduction is determined by the steady state fraction of reduced electron acceptors, FeS and heme *b*_L_ in CIII_2_. Assuming that the *E*_m7_ values of these electron acceptors are the same in *M. smegmatis* as in *C. glutamicum*^17^, oxidation of MQH_2_ in *M. smegmatis* is slightly exergonic, but would become endergonic with UQH_2_, which would result in a smaller fraction of reduced initial acceptors and thereby a lower activity. Hence, since in the current experiments DCQH_2_ and DMNQH_2_ compete for binding to the same (Q_o_) site, the difference in their *E*_m7_ values would explain the difference in turnover numbers. Yet another possibility is that, in contrast to DMNQH_2_, DCQH_2_ is stabilized at the more distant MQ_o1b_ position and does not move to MQ_o1a_, which is required to allow electron transfer to the FeS center. Qualitatively, the same explanation would explain the decrease in activity upon addition of DCQH_2_ to cytochrome *bd*-I, but we were not able to establish whether or not DCQH_2_ and DMNQH_2_ bind to the same site in this enzyme. Inhibition by ubiquinol of the *M. tuberculosis* cytochrome *bd* was also recently indirectly observed^52^.

As previously mentioned, targeting respiration with antimycobacterial drugs is complicated because of the branched respiratory chains of actinobacteria, which also adapt to environmental conditions^36,43,53^. In other words, more potent killing of these bacteria would require inhibition of both branches^43,54,55^. Identifying inhibitors of mycobacterial respiration is also challenging due to the complexity and redundancy of the *M. tuberculosis* respiratory chain, which allows the bacterium to adapt to various stress conditions^56^. The pathogen’s metabolic heterogeneity and ability to persist in different physiological states further complicate drug targeting. Additionally, ensuring that inhibitors are effective within the hostile intracellular environment of macrophages, while remaining selective enough to avoid host mitochondrial toxicity, is a major hurdle. Resistance development and limitations in current screening assays also pose significant obstacles to discovering effective and safe respiratory inhibitors.

Earlier studies demonstrated partial inhibition of *M. smegmatis* growth when the CIII_2_CIV_2_ respiratory branch was blocked^57^, supporting the feasibility of potential drug candidates such as telacebec (Q203) or lansoprazole sulfide (LPZS)^10,37,40–42^. In the current study, we observed an almost complete growth inhibition of *M. smegmatis* in the presence of DCQ, although inhibition of the CIII_2_CIV_2_ respiratory branch by DCQ occurred at higher IC_50_s compared to those of e.g., Q203 or LPZS^37^. Remarkably, DCQ also inhibited the growth of virulent *M. tuberculosis* bacilli in human macrophages, which suggests that the compound may be used as an adjuvant during treatment of tuberculosis (TB) disease. The mechanism of this inhibition is presently unknown, but it is possibly related to the effect of DCQ on DMNQH_2_ oxidation: O_2_ reduction activities of both the CIII_2_CIV_2_ supercomplex and cytochrome *bd* branches. A previous study reported that coenzyme Q10 stimulated the growth of *M. tuberculosis* H37Rv^58^, which contrasts sharply with our findings that DCQ inhibits *M. tuberculosis* growth. However, the experimental conditions differed significantly. The earlier work assessed planktonic *M. tuberculosis* growth in liquid medium supplemented with both pyruvate and succinate, and observed growth stimulation only when both substrates were present^58^. In contrast, our study investigated intracellular *M. tuberculosis* growth within human monocyte-derived macrophages at a low MOI, a model that more closely reflects physiological conditions. Notably, our culture medium (RPMI) did not contain succinate. Although we did not directly assess the effects of DCQ on human macrophages in this study, it is also plausible that appropriate doses of DCQ may support immune cell function. Native coenzyme Q10 has been reported to enhance phagocytic and antimicrobial activity, improve mitochondrial energy production, and exhibit antioxidant and anti-inflammatory properties, and support of lysosomal function^59,60^. Similarly, its analog DCQ has been shown to reduce reactive oxygen species (ROS) production in human cells and mitigate oxidative stress in rat serum samples^61^. DCQ has also demonstrated a potent ability to inhibit angiogenesis^62^, which may interfere with the chronic inflammatory milieu^63^ that is central in TB pathogenesis. As such, these mechanisms could contribute to improved control of intracellular *M. tuberculosis* growth.

While coenzyme Q10 is generally regarded as safe and beneficial at physiological or therapeutic concentrations^60^, the use of coenzyme Q10 or DCQ at elevated doses in cell culture may raise concerns, including oxidative stress, mitochondrial dysfunction, altered cell signaling, and potential disruption of lysosomal integrity^61,64,65^. Accordingly, our results indicate that both DCQ and DNMQ can compromise host cell integrity at higher concentrations. However, we did not observe such adverse effects within the lower dose range of DCQ, up to 100 µM. Notably, these lower concentrations were equally effective in reducing *M. tuberculosis* growth compared to the higher dose of 250 µM. In macrophage-based cell culture experiments, DCQ is typically used at concentrations ranging from 1 µM to 100 µM. Further investigations are warranted to explore the potential benefits as well as adverse effects of DCQ on the viability and function of *M. tuberculosis*-infected macrophages across a range of concentrations.

As ubiquinone plays a central role in the mitochondrial electron-transfer chain, therapeutic regimens that target bioenergetics have shown promising potential to enhance bactericidal activity against *M. tuberculosis*, with the added prospect of shortening treatment duration^66^. UQ analogs or modulators could contribute to such approaches, particularly if bacterial respiration is selectively impaired without harming host cells. Some anti-TB drugs, such as bedaquiline, directly target components of the bacterial electron-transfer chain, while others, like rifampicin, isoniazid and streptomycin, interfere with cell wall synthesis and protein translation. Combining agents that disrupt different aspects of *M. tuberculosis* physiology, including electron-transfer chain components, can result in additive or synergistic killing effects. Coenzyme Q10 and its analogs may enhance the efficacy of existing anti-TB drugs not only by targeting *M. tuberculosis* bioenergetics but also by supporting host immune responses, reducing pathological inflammation and reducing drug-induced host toxicity^59,60,67–69^. Coenzyme Q10 has also been shown to improve mitochondrial and lysosomal function as well as phagocytic activity in macrophages^59,70,71^, potentially boosting antimicrobial activity^60,72^. Its antioxidant properties may contribute to protecting host cells from the cytotoxic effects associated with certain TB drugs^60^, thereby contributing to more effective and better-tolerated combination therapies. It remains unclear whether DCQ retains these beneficial effects in Mtb-infected immune cells. Further studies will deepen our understanding of the mechanistic action of DCQ to block mycobacterial respiration and uncover potential synergistic or additive effects with conventional antibiotics for TB treatment.

## Materials and methods

### Mycobacterial strains and cultures

*M. smegmatis* mc^2^155 cells transformed with qcrCAB/pST-KT (C-terminally FLAG-tagged on QcrB), were grown on 7H10 ^73^ agar plates supplemented with 10% albumin-dextrose-saline (ADS) and antibiotics (25 μg ml^−1^ kanamycin) at 37 °C. Single colonies were picked from the agar plates, inoculated into 25 ml culture, and shaken at 180 rpm, 30 °C. The pre-cultures were diluted after 48 h into 1 L 7H9 medium in a 2-L flask, and shaken at 180 rpm at 30 °C for ~72 h. The 7H9^73^ medium was supplemented with ADS, 25 μg ml^−1^ kanamycin, 50 μg ml^−1^ hygromycin, 0.2% glycerol, and 0.05% Tween 80. Cells were harvested (6000 x *g*, 30 min; JLA 8.1 rotor (Beckman Coulter^TM^)) when the OD_600_ reached 2.

*M. smegmatis* mc^2^155 cells transformed with pST-K*cydAB*_TwinStrep_ (Strep-tagged on CydB) were grown essentially the same way, except that the large cultures were in 2 L medium in 2.8 L flasks.

*M. tuberculosis* laboratory strains H37Ra (avirulent) or H37Rv (virulent), transfected with a plasmid carrying a gene encoding GFP (pFPV2), were from ATCC (Rockville, MD). Sub-cultures of mycobacteria in Middlebrook 7H9 media supplemented with 10% Oleic Albumin Dextrose Catalase (OADC), 0.05% Tween-80, and 20 µg/ml kanamycin (Karolinska University Hospital, Stockholm, Sweden) were incubated at 37 °C for 2 weeks, after which an aliquot was transferred to fresh growth medium for an additional week of expansion. Mycobacterial cultures were washed in 0.05% PBS-Tween-80 followed by pulse-chase sonication to disrupt bacterial clumps. The optical density of the bacterial suspension was measured at 600 nm and adjusted to 0.2–0.3, then used to infect human monocyte-derived macrophages as described below.

### Growth of *M. smegmatis* on plates supplemented with DCQ/DMNQ

The effect of Decylubiquinone (DCQ, CAS no. 55486-00-5) and 2,3-Dimethyl-1,4-naphthoquinone (DMNQ, CAS no. 2197-57-1) on the growth of *M. smegmatis* cells on solid LB agar was tested by inoculating a 5 ml liquid LB medium with a single colony of *M. smegmatis* mc^2^155 strain and growing it for two days at 37 °C with constant shaking at 180 RPM. After 2 days, a series of dilutions was prepared using a liquid LB medium ranging from no dilution to a dilution by a factor of 10^5^. Stocks of 50 mM DCQ/DMNQ were prepared by dissolving 16 mg of DCQ powder (Sigma) in 1 ml ethanol (99.45% ethanol, Sigma) or 16 mg of DMNQ powder (Sigma) in 2 ml ethanol. Subsequently, plates containing either DCQ or DMNQ were prepared by adding a fixed volume of DCQ/DMNQ stocks (7.5 μl for 25 μM, 15 μl for 50 μM) to 15 ml of LB + agar (per plate) before it solidified. Finally, 5 μl of each dilution was plated on the corresponding part of the plate and grown for another two days at 37 °C.

### *M. smegmatis* membrane preparation

For preparation of the CIII_2_CIV_2_ supercomplex, cells were homogenized in cell lysis buffer (50 mM Tris-HCl, pH 7.5, 50 mM NaCl, 0.5 mM EDTA) in the presence of phenyl-methanesulfonyl fluoride and DNase I (Roche), and crushed with a cell disrupter (Constant Systems Ltd.) with four cycles at 35 kPsi. Debris material was removed by centrifugation at 18,600 × *g* for 30 min. This step was followed by membrane separation using ultracentrifugation at 147,000 × *g* for 90 min.

For preparation of cytochrome *bd*-I, approximately 50 g of mc^2^155 pST-K*cydAB*_TwinStrep_ cells (wet weight) were homogenized in 300 ml of 50 mM HEPES at pH 7.4, 150 mM NaCl, in the presence of one tablet of cOmplete^TM^ Mini Protease Inhibitor Cocktail (Roche) and a small amount of DNase I. Cell disruption was performed through three to four passes at 35 kpsi using a cell disruptor (Constant Systems Ltd.), followed by centrifugation to pellet cell debris at 15 500 × *g* for 15 min. The resulting cell membranes were then separated via ultracentrifugation at 215,000 × *g* for 90 min.

### Isolation of *M. smegmatis* supercomplexes and cytochrome *bd*

To isolate the CIII_2_IV_2_ supercomplex, membranes (1 g) were mixed with 10 ml of a solubilization buffer 50 mM Tris-HCl, pH 7.5, 100 mM NaCl, 0.5 mM EDTA, 2% (w/v) GDN (Anatrace) and incubated at 4 °C for 24 h under stirring. Insolubilized debris material was removed by ultracentrifugation at 147 000 × *g* for 30 min. The supernatant was then applied to a previously equilibrated anti-FLAG M2 column (1 ml, Sigma Aldrich). The column was washed with four column volumes of washing buffer containing 50 mM Tris-HCl, pH 7.5, 100 mM NaCl, 0.5 mM EDTA, 0.01% (w/v) GDN. Five column volumes of elution buffer composed of the washing buffer supplemented with 5 mg ml^−1^ FLAG peptide (Sigma Aldrich) were used to elute the protein, which was further concentrated with a 100-kDa molecular weight cut-off concentrator (Merck Millipore). The protein samples were aliquoted, flash frozen in liquid N_2_ and stored at −80 °C until use.

To isolate cytochrome *bd*-I, harvested membranes (typically 5–10 g) were resuspended in 50 mM HEPES at pH 7.4, 150 mM NaCl to achieve a final protein concentration of 10 mg/ml, and were solubilized by the addition of 1% (w/v) N-Dodecyl-β-D-maltoside (DDM) with gentle stirring for 60 min. Any insolubilized material was removed by centrifugation at 215,000 × *g* for 60 min, after which the supernatant was subjected to affinity chromatography. The solubilized protein was then applied to a gravity column (2 ml Strep-tactin Superflow Plus, VWR) that had been pre-equilibrated with a buffer composed of 50 mM HEPES at pH 7.4, 150 mM NaCl, and 0.05% DDM. After sample loading, the column was washed with 10 ml of the buffer used for pre-equilibration of the column and the elution was performed using 10 ml of the same buffer supplemented with 10 mM desthiobiotin.

### Activity measurements

The activity of the purified *M. smegmatis* supercomplex (O_2_ reduction: MQH_2_ oxidation) was measured by following the O_2_ reduction rate upon addition of reduced 2,3-dimethyl-[1,4] naphthoquinone (DMNQH_2_) (Rare Chemicals GmbH) using a Clark-type oxygen electrode (Hansatech instruments). The reaction was started by the addition of 5 μL of 20 mM DMNQH_2_ into an electrode chamber containing 1 ml protein solution in a buffer composed of 50 mM Tris-HCl, pH 7.5, 100 mM NaCl, 0.5 mM EDTA, 0.01% (w/v) GDN at 25 °C. The activity was obtained from the initial slope of the graph, where the O_2_ concentration was linearly dependent on time. DMNQH_2_ is spontaneously oxidized by O_2_, but the auto-oxidation rate decreases upon addition of 0.5 μM bovine superoxide dismutase (SOD), which was used routinely in our measurements according to the assay described in detail in ref. ^10^. We first monitored the O_2_-reduction rate by DMNQH_2_ before addition of the supercomplex and this slope was subtracted from that obtained after addition of the supercomplex. The difference reflects the quinol oxidation: O_2_ reduction activity of the supercomplex^10^.

The oxidoreductase activity of cytochrome *bd*-I was measured in a solution composed of 50 mM HEPES pH 7.4, 150 mM NaCl, 0.05% DDM at 25 °C with the use of a Clark-type electrode (Hansatech instruments). Reduced DMNQH_2_ was used as the substrate. A volume of 2.5–15 μL of a 20 mM stock solution of reduced DMNQH_2_ was added in order to monitor the background oxidation rate. The reaction was then initiated by addition of cytochrome *bd*-I (~100 nM). Reduction of DMNQ and DCQ were performed as described in ref. ^8^. Briefly, quinone was dissolved in ethanol to yield a 20 mM solution. Several crystals of sodium borohydride (NaBH_4_) were added to reduce the quinone to quinol. The solution was kept on ice until transparent and HCl was added until formation of small bubbles in the solution ended. The sample was centrifuged for 10 min at 10,000 x *g*, and the supernatant containing reduced quinol was aliquoted, flash frozen, and stored in −80 °C until use.

### *M. tuberculosis* macrophage infection model

Buffy-coat blood was obtained from healthy adult donors at the Karolinska Hospital Blood Bank, Stockholm, Sweden, with informed consent and ethical approval (2010/603-31/4). Lymphoprep™ (Alere technologies, Norway) is a density gradient medium that was used for the isolation of mononuclear cells from peripheral blood. Monocytes were separated from bulk mononuclear cells using 2–3 h plastic adherence in T75 flasks (Thermo Fisher Scientific, Waltham, MA) or 6-well plates at 37 °C in serum-free media (RPMI 1640, VWR, Radnor, PA). Non-adherent cells were washed off, and Macrophage colony-stimulating factor (M-CSF, 50 ng/ml) was supplemented to the cell culture media (RPMI including 10% fetal calf serum (FCS), 2 mM L-glutamine, 1 mM sodium pyruvate, 5 mM HEPES) to allow differentiation of monocytes to macrophages for 7 days. Human monocyte-derived macrophages were detached from the cell culture flasks or plates using 2 mM EDTA buffer including 2.5% FCS for 30 min at 37 °C and used in experiments as described below.

### High-content imaging with IncuCyte

For high-content analysis of macrophages with IncuCyte, cells from two healthy donors (*n* = 2) were prepared in T75 flasks and re-seeded at a density of 4000 cells/well in optilux Black/Clear Flat bottom, TC-treated 384-well plates (Falcon Corning, Corning, NY). Cell cultures were infected with H37Rv-GFP at a multiplicity of infection (MOI) of 1 and treated with DCQ or DMNQ in triplicates. The untreated MOI1 infection control was cultured in medium with diluent only. Mycobacterial growth (green fluorescent signal) and cell morphology were assessed using high-content imaging with the IncuCyte® S3 Live-Cell Analysis System (Sartorius, Göttingen, Germany) after 4 days of incubation of the plates at 37 °C. Image acquisition was based on a single image of the whole well that was captured using a 4× objective (original magnification) at a single time point on day 4 post-infection. The fluorescent signal from the mycobacteria was quantified as total integrated intensity (GCU x µm2/image) using the IncuCyte S3 software. Data (median and range) from *n *= 2–4 donors was plotted and analyzed in GraphPad Prism 9 (version 9.5.1) as the percent (%) intracellular *M. tuberculosis* growth in macrophages relative to the untreated MOI infection control (set to 100%). IncuCyte results were analyzed using a 2-way ANOVA and Tukey’s multiple comparisons test to calculate the indicated p-values.

### Flow cytometry

For flow cytometry analysis of macrophages, cells from two healthy donors (*n* = 2) were cultured in 6-well plates at a density of 1 million cells per well (Falcon Corning). Cell cultures were infected with H37Ra-GFP at an MOI of 5 for 4 h, followed by washing and treatment with either DCQ or DMNQ. Untreated *M. tuberculosis*-infected macrophages cultured in medium with diluent only served as positive controls, while uninfected macrophages were used as negative controls. After 48 h of incubation, the cells were washed with FACS buffer (PBS, 2% FCS, 0.05 mM EDTA) and stained for 15 min with LIVE/DEAD Near IR Viability Dye (APC-Cy7) (Thermo Fisher Scientific). An Fc-blocking cocktail (Miltenyi, Germany) was used to minimize nonspecific binding. Red blood cells were lysed using a fixation/permeabilization working solution (BD Cytofix/Cytoperm™, Franklin Lakes, NJ), and the cells were resuspended in FACS buffer. All stained samples were acquired on an LSR Fortessa (BD Biosciences) at low to medium speed. Macrophages infected with H37Ra-GFP bacteria were detected in the FITC channel during flow cytometry analysis (see Supplementary Fig. S1**)**. Gating strategies included singlet isolation (to exclude doublets) and viability gating (to exclude dead cells). The fluorescence intensity of the GFP signal was visualized in a histogram and quantified as the geometric mean (GM).

### Cryo-EM sample preparation and data collection

GDN solubilized purified supercomplex protein sample (3 μl of 0.5 mg/ml) was mixed with 30 μL 200 mM stock solution of DCQ, incubated for 5 min at room temperature and concentrated to a final concentration of 3.5 mg/ml. This DCQ-containing sample was used for cryo-EM experiments. Copper EM grids coated with holy carbon film (300 mesh R2/2 grid, Quantifoil, Micro Tools GmbH, Germany) were used for the sample preparation. The grids were glow-discharged in air at 20 mA for 120 s (PELCO easiGlow). Vitrobot Mark VI (Thermo Fisher Scientific) was used for the sample blotting (2 μL of the protein, 3 s at 4 °C, 100% humidity) and plunge-freezing in liquid ethane. Titan Krios G3i electron microscope (Thermo Fisher Scientific) Ceta-D camera and a Gatan K3 BioQuantum detector was used for data collection. The dataset was acquired in electron-counting mode at a nominal magnification of 10,5000 (0.825 Å/pixel) and contained 15,000 exposures (40 exposure fractions each). EPU software package (v2.12.1; Thermo Fisher Scientific) was used for automated data collection with camera exposure rate was 16.5 e^−^/pixel/s and total exposure of the sample 40 e^−^/Å^2^ (Supporting information Supplementary Fig. S2 and Table 1).

**Table 1:** Data collection and processing of cryo-EM data

Data collection, processing	UQ
PDB code	9QEF
Voltage (kV)	300
Magnification	105000
Electron exposure (e^−^/Å^2^)	40
Pixel size (Å)	0.825
Defocus range (μm)	−2.2 to −0.6
Defocus step (μm)	−0.2
Symmetry imposed	None C1
Initial particle images (number)	4 072098
Final particle images (number)	79000
FSC threshold	0.143
Map resolution (Å)	2.5
Refinement	
CC (mask)	0.88
Resolution estimates (Å)	
d 99	
Masked	3.1
Unmasked	3.0
d FSC model, 0/0.143/0.5 (Å)	
Masked	2.3/2.5/2.9
Unmasked	2.5/2.5/3.1
Model composition	
Protein residues	5797
Non-hydrogen atoms	49157
Ligands	84
Water	606
B factors (Å^2^)	
Min/max/mean	
Protein residues	6.12/190.06/82.59
Ligands	5.67/176.09/81.47
water	54.44/137.02/76.92
Validation MolProbity	
Ramachandran plot	
Favored (%)	97.28
Allowed (%)	2.71
Disallowed (%)	0.02

### Cryo-EM data processing, structure building, and refinement

CryoSPARC v4^74^ was used for the processing of the dataset, with motion correction and CTF estimation performed using the Patch motion correction and Patch CTF estimation, as implemented in cryoSPARC. Initial particles used for the generation of initial 2D classes, later used by the Template picker job, were picked from 1000 images using the Blob picker job in cryoSPARC. The initial set of particles (4,072,098) was reduced by 2D classification to 1,521,965 particles, of which 55,9531 were selected for another round of 2D classification. Resulting group of 187,202 particles was used for ab initio reconstruction set for 3 classes. The best class of ab initio reconstruction contained 99,481 particles, which were re-extracted from micrographs with box size 540 px without Fourier crop. The reextracted particles were separated into another two classes using ab initio reconstruction. The best class contain final set of 79,220 particles. These were further refined using homogenous refinement, followed by local CTF refinement without symmetry enforced and a second round of homogenous refinement. The resolved final map had an overall resolution of 2.54 Å. The initial structure model used for map interpretation was from a previously solved structure of the supercomplex with the PDB code 8OVD^42^. The model was refined against the map using Coot^75^ and real-space refinement in Phenix^76^.

### Statistical analysis and reproducibility

Statistical differences comparing different doses of compounds (median ± range) were determined using a 2-way ANOVA and Tukey’s multiple comparisons test. All values are presented as mean ± SD, derived from at least three independent experiments.

### Reporting summary

Further information on research design is available in the Nature Portfolio Reporting Summary linked to this article.

## Supplementary information

Supplementary Information

Description of Additional Supplementary Files

Supplementary Data 1

Reporting Summary

## Data Availability

Structural data generated in this study have been deposited in the EMDB and PDB databases under the following accession codes: EMD-53065 and 9QEF. The cryo-EM raw images are available from authors upon request. Activity data are found in Supplementary Data 1.
