# Supplementary material for: Mycobacterial respiratory chain enzymes and growth are inhibited by decylubiquinone
Source: Commun Biol. 2025 Dec 10;9:43. doi: 10.1038/s42003-025-09309-9 (PMC12789660; doi:10.1038/s42003-025-09309-9)
Supplement: Supplementary file 1 — Supplementary Information [file 42003_2025_9309_MOESM1_ESM.pdf]

# Supporting information for

# Mycobacterial respiratory chain enzymes and growth are inhibited by decylubiquinone

Sylwia Król[1#], Terezia Kovalova[1#], Mateusz Janczak[1], Sadaf Kalsum[2], Mira Akber[3], Martin Högbom[1], Susanna Brighenti[3], Pia Ädelroth[1] and Peter Brzezinski[1]*

[1]Department of Biochemistry and Biophysics, The Arrhenius Laboratories for Natural Sciences, Stockholm University, 106 91 Stockholm, Sweden

[2] Division of Medical Microbiology and Molecular Medicine, Department of Clinical and Experimental Medicine, Linköping University, Linköping 581 83, Sweden

[3] Center for Infectious Medicine (CIM), Department of Medicine Huddinge, Karolinska Institutet, ANA Futura, Huddinge 141 52, Sweden

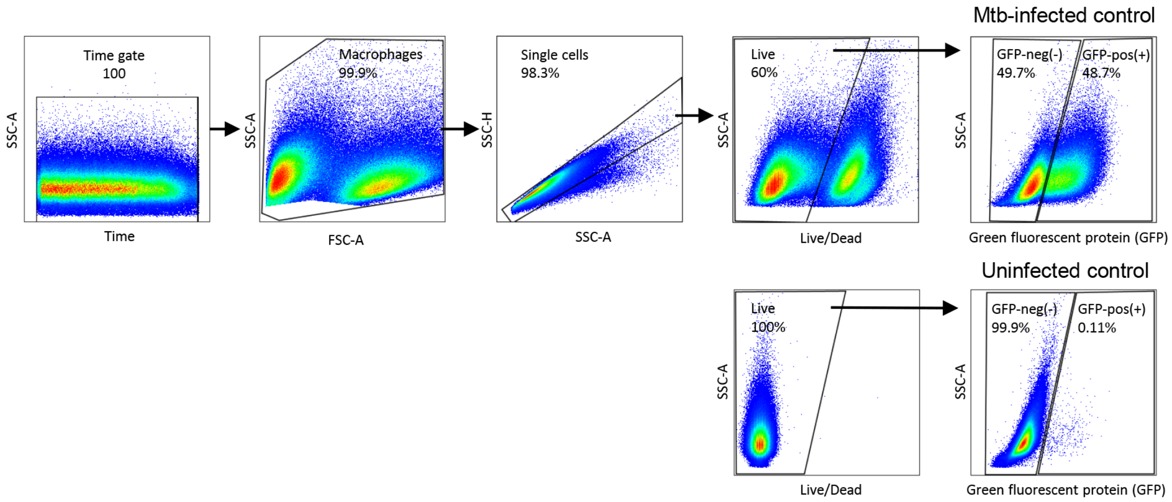


**Supplementary Figure S1. Gating strategy on monocyte-derived macrophages using flow cytometry**. Flow cytometry was used to analyze in vitro differentiated macrophages, either uninfected or infected with green fluorescent protein (GFP)-expressing *M. tuberculosis* H37Rv. Initial time gating was applied to detect and exclude acquisition artifacts. Next macrophages were identified using forward scatter (FSC) versus side scatter area (SSC-A) dot plots. Singlets were selected by gating SSC-H versus SSC-A to eliminate doublets, followed by viability gating using SSC-A versus a Live/Dead marker (Zombie-UV, Invitrogen) to exclude non-viable cells. From the resulting live singlet population, macrophages were further gated based on GFP expression. Gates were applied to distinguish GFP-negative and GFP-positive macrophages. Histograms presented in the main manuscript were generated from the GFP-positive live cell population. This gating strategy was consistently applied across all samples in the study. Representative dot plots from one donor illustrate the workflow for both Mtb-infected and uninfected macrophages.

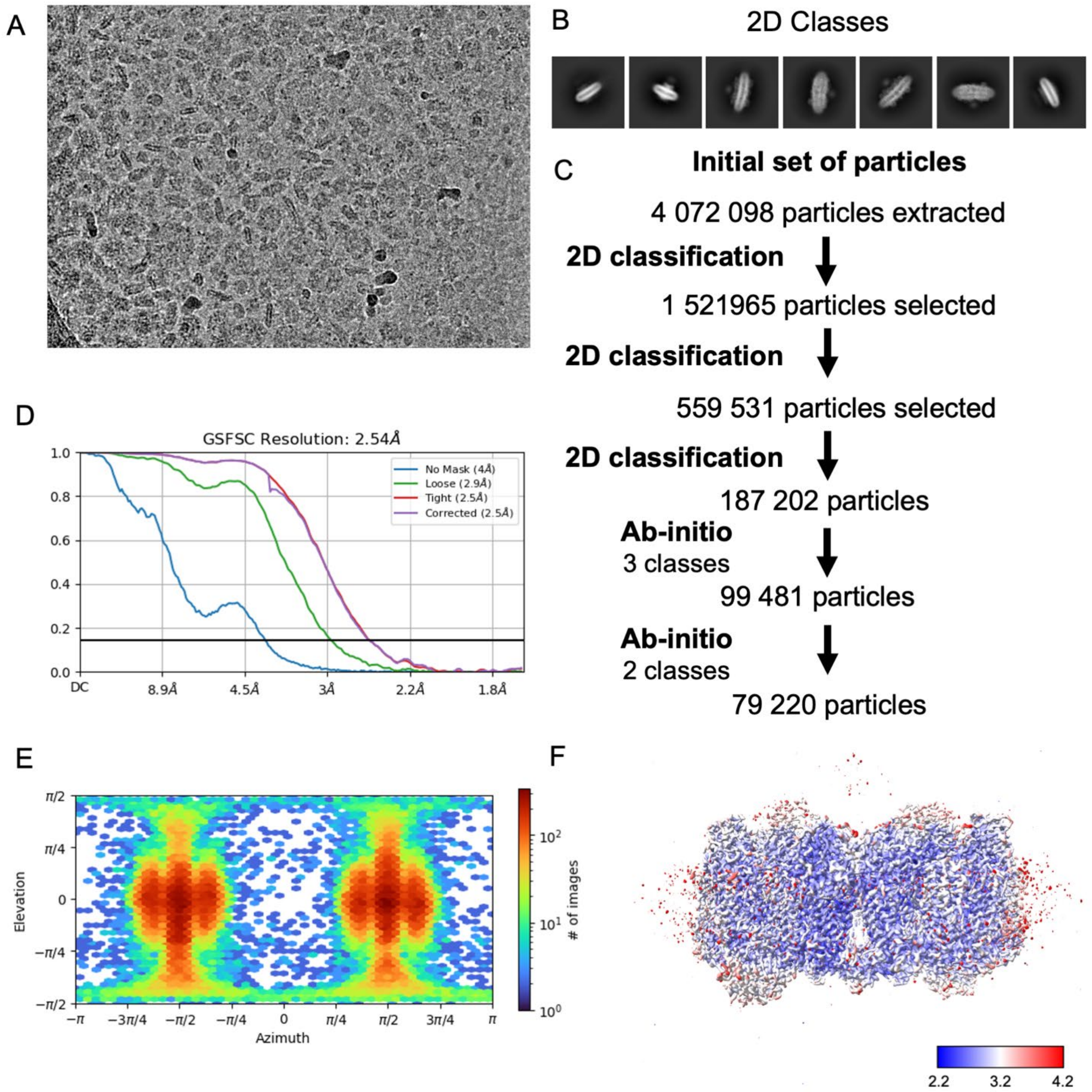


**Supplementary Figure S2. Cryo-EM validation.** (**A**) Example micrograph. (**B**) 2D class averages. (**C**) Workflow for cryo-EM image analysis. (**D**) Fourier shell correlation (FSC) curve after refinement, corrected for the effects of masking. (**E**) Viewing direction distribution for particle images. (**F**) Local resolution map (numbers in units of Å).
