# Supplementary material for: Mycobacterial respiratory chain enzymes and growth are inhibited by decylubiquinone
Source: Commun Biol. 2025 Dec 10;9:43. doi: 10.1038/s42003-025-09309-9 (PMC12789660; doi:10.1038/s42003-025-09309-9)
Supplement: Supplementary file 2 — Description of Additional Supplementary Files [file 42003_2025_9309_MOESM2_ESM.pdf]

Description of Additional Supplementary files

File name: Supplementary Data 1

Description: Activity data are found in Supplementary Data 1.
